# The Effectiveness of Subepithelial Connective Tissue Graft as a Barrier Membrane in Guided Bone Regeneration: A Case Report

**DOI:** 10.7759/cureus.48741

**Published:** 2023-11-13

**Authors:** Ahmed M Kabli

**Affiliations:** 1 Department of Preventive Dental Sciences, College of Dentistry, Taibah University, Madinah, SAU

**Keywords:** ridge augmentation, case report, tissue contour, barrier membrane, subepithelial connective tissue graft

## Abstract

Subepithelial connective tissue graft (SCTG) can be used as a membrane in guided bone regeneration (GBR) to gain more tissue volume. The case presents a 66-year-old female patient with a missing canine and is referred for dental implant placement. The implant was placed; GBR was done using xenograft particulate and a palatal SCTG as a membrane to improve the labial contour and achieve better esthetics. She returned for a routine follow-up after 14 months, and the examination revealed a well-maintained tissue contour following the final crown placement.

## Introduction

Guided bone regeneration (GBR) is a common surgical procedure that has been used to increase bone volume or reconstruct lost bone in edentulous ridges and around dental implants, so they can be placed in the desired restoratively driven position and proper diameter. The concept of the procedure is to use a barrier membrane to prevent soft tissue invasion with or without bone graft [1].

The membranes must meet specific criteria, including biocompatibility, space maintenance, and easy manipulation [2]. There are two types of membranes, resorbable and non-resorbable, with each having its characteristics that help in the decision of the type to be used. However, in some cases where there are soft and hard tissue defects, especially in esthetic areas, subepithelial connective tissue graft (SCTG) can be used as a membrane to enhance the thickness of the soft tissue since available membranes are thin.

The available literature showed that palatal SCTG can be effective as an autogenous barrier membrane [3]. However, only a few studies used SCTG as a membrane in GBR to graft the labial or buccal aspect of dental implants.

This case report presents the clinical outcomes of a case that was treated with a dental implant using SCTG as a barrier membrane in GBR in an attempt to improve the labial ridge contour and esthetics around the placed dental implant. The case has a total follow-up period of 14 months.

## Case presentation

**Figure 1:**
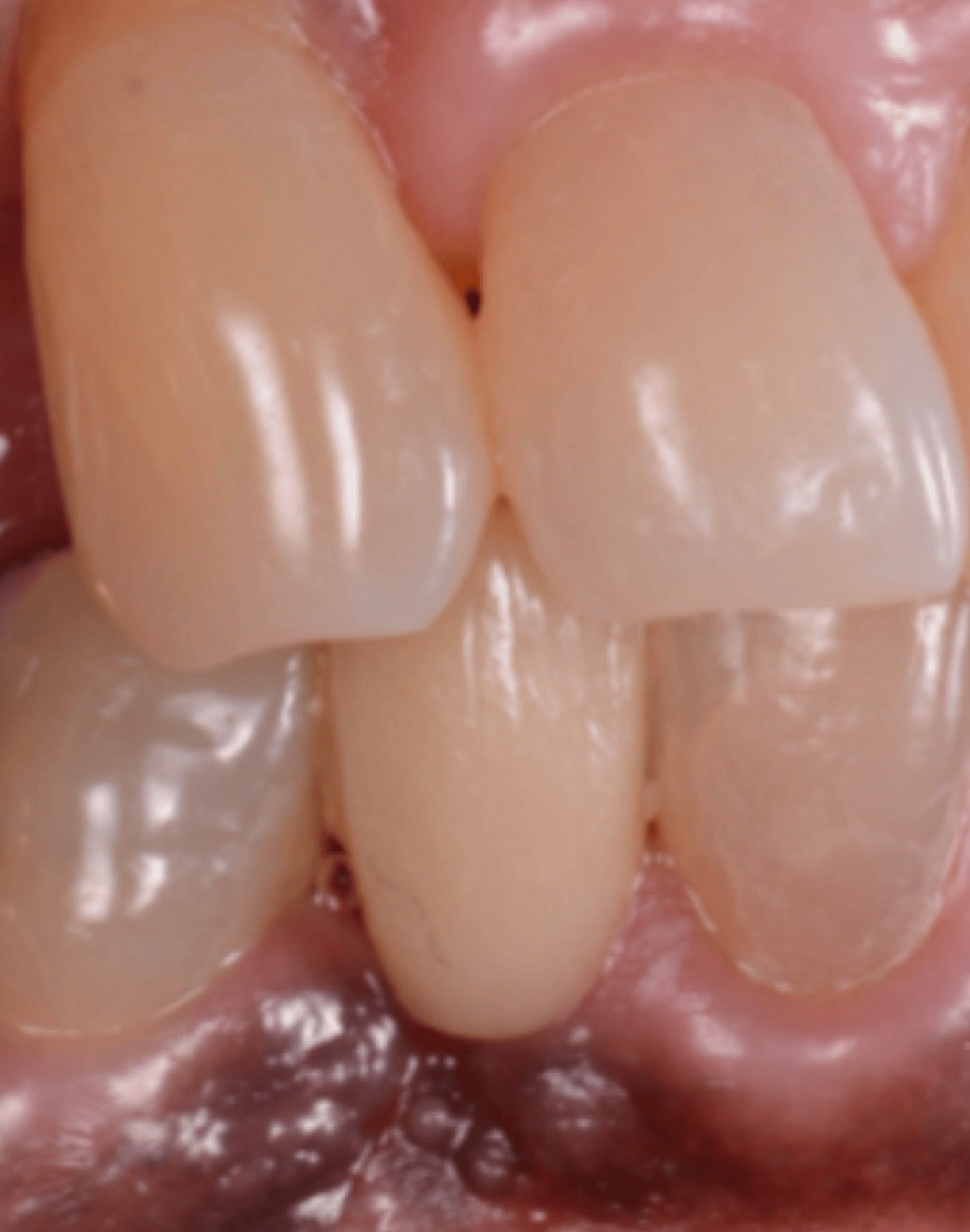
Intraoral picture showing the tissue defect in the lower canine area

A 66-year-old female patient came to the dental clinic to replace a missing mandibular right canine with an implant. The missing tooth area had a cantilever bridge using the mandibular right first premolar as an abutment (Figure 1).

**Figure 2:**
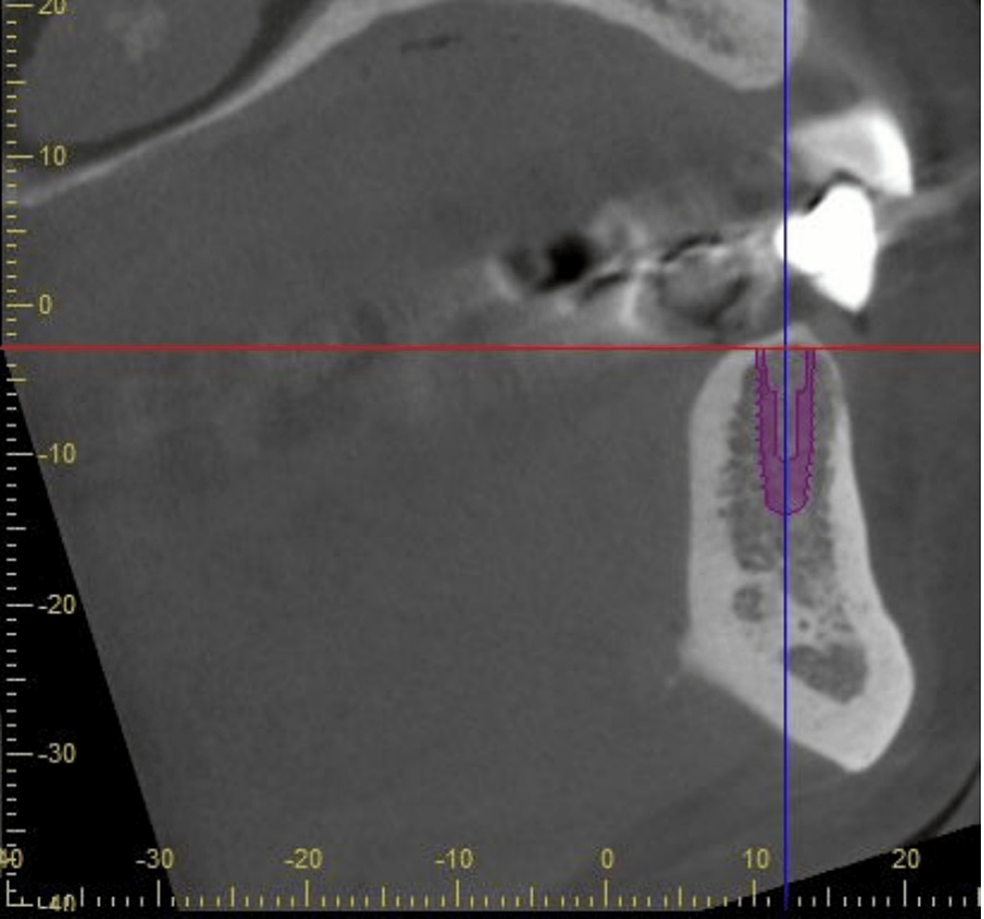
Sagittal view of pre-operative CBCT demonstrating implant planning CBCT: cone-beam computed tomography

The referring dentist planned to replace the bridge with a new crown on the mandibular right first premolar and replace the missing mandibular right canine with a single dental implant. Clinical evaluation revealed horizontal ridge deficiency at the canine site. The radiographic examination did not reveal any pathological lesions. After obtaining the consent, a cone-beam computed tomography was obtained, and the bridge was removed to assess the restorability (Figure 2).

The referring dentist placed the temporary bridge before referring the case to the periodontics clinic. After the clinical examination and the review of the radiographs, it was decided to graft the canine labially and use an SCTG as a membrane to bulk out the area and improve esthetics.

**Figure 3:**
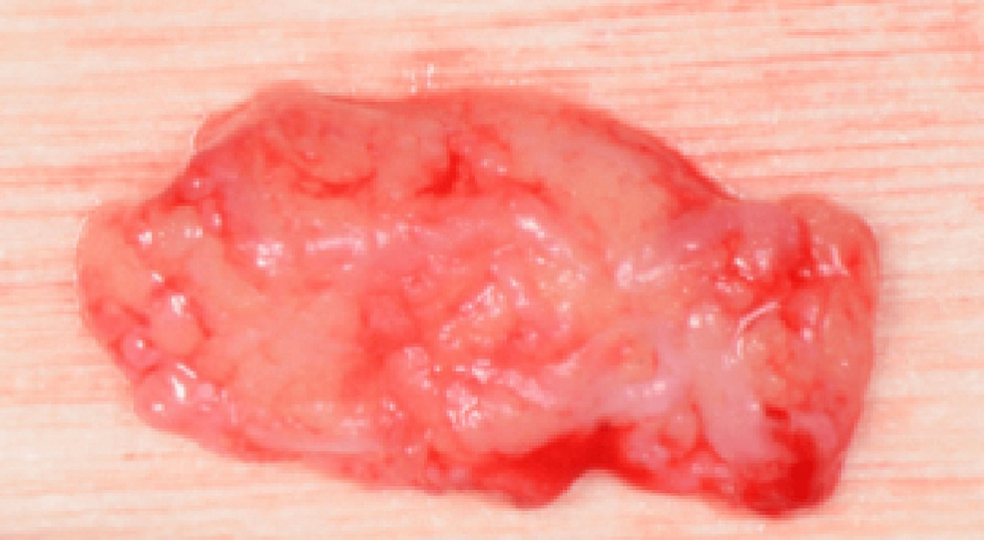
The harvested 11×6 mm palatal SCTG SCTG: subepithelial connective tissue graft

After infiltration with 2% lidocaine local anesthesia, a crestal incision was done using a 15C blade, and full-thickness mucoperiosteal flaps were reflected labially and lingually. A 4/3×11.5 mm platform-switched tapered implant (Biomet 3i, Palm Beach Gardens, FL) was placed, and primary stability was achieved. An 11×6 mm SCTG was harvested from the palate (Figure 3).

**Figure 4:**
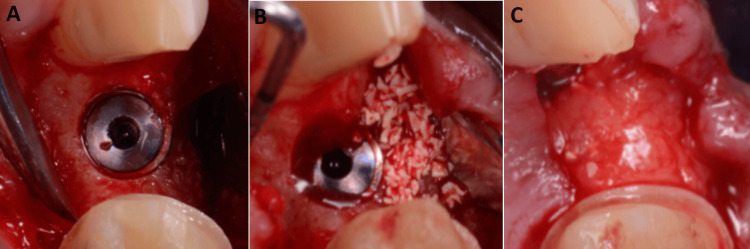
(A) Dental implant placement, (B) xenograft application, and (C) SCTG membrane SCTG: subepithelial connective tissue graft

Xenograft particulates were packed labially, and the SCTG was used as a membrane extending from the labial aspect to cover the bone graft and the implant (Figure 4).

Horizontal mattress and simple interrupted sutures using 5-0 polypropylene were placed, and the temporary bridge was recemented again after relieving the canine area. Post-operative instructions were given to the patient, and she was scheduled for post-operative evaluation.

**Figure 5:**
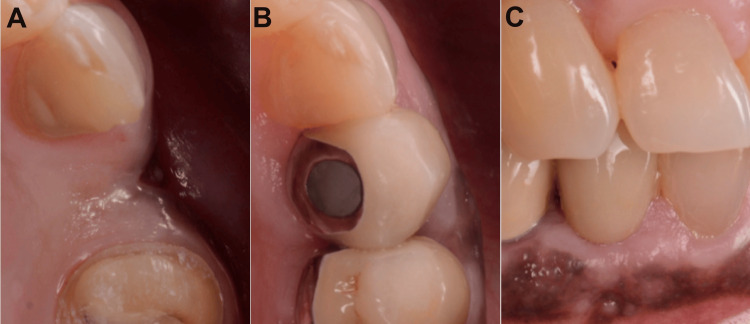
(A) Occlusal view of the pre-operative clinical picture and (B) occlusal and (C) labial view at 14-month post-operative follow-up

At two weeks, healing was uneventful, and all sutures were removed. At the three-month recall visit, clinical and radiographic examinations showed good esthetics and osseointegration. Second-stage surgery was done to replace the cover screw with the healing abutment. Two weeks after the second-stage surgery, an assessment was done to ensure soft tissue healing around the healing abutment, and the patient was referred to the restorative dentist. She returned for a routine follow-up after 14 months, and the examination revealed a well-maintained tissue contour following the final crown placement (Figure 5).

Ethical approval was received from the ethics committee of the College of Dentistry, Taibah University (TUCDREC/151023/AKabli).

## Discussion

It has been shown that ridge remodeling occurs after teeth extraction, leading to dimensional ridge changes. These dimensional changes appear more in the buccal than the palatal or lingual side of the ridge [4]. Other factors known to cause changes in the width of the ridge, such as trauma and lesions, must be noted. To compensate for these changes, GBR is frequently done to correct the contour and increase the width of the ridge, thus providing a better site for implant placement and better esthetics [5]. The basic principle of doing a GBR procedure is to use a barrier membrane to prevent the non-osteogenic cells from migrating to the site, and it can be done with or without bone graft [6].

Different types of barrier membranes are widely used with different techniques depending on multiple factors such as the membrane's physical properties, type of surgery, or operator preference [7]. For these membranes to be ideal, they should be easy to manipulate with good mechanical and biological properties and have bioactive and antibacterial properties while also acting as a tissue barrier [8].

The literature revealed that palatal SCTG can be successfully used as a barrier membrane [3]. The gingival connective tissue was shown to have the ability to differentiate into osteogenic cells [9]. Additionally, using SCTG can enhance the bulk of the soft tissue and improve the esthetics. It is also easily available, adaptable, and cost-effective [10]. On the other hand, it needs a second surgical site and more surgical time and causes more discomfort to the patient.

In this case, we successfully improved the soft tissue contour and esthetics at the implant site by grafting the site labially with xenograft and using palatal SCTG as a barrier membrane. The result was stable at a 14-month post-operative follow-up and after the final restoration placement.

## Conclusions

Guided bone regeneration (GBR) is a widely performed procedure with the increased use of dental implants to restore missing teeth. The subepithelial connective tissue graft (SCTG) can be successfully used as a barrier membrane in GBR around dental implants. In this case, the SCTG increased the tissue volume and had stable results over 14 months. In addition, it corrected the tissue contour and improved the esthetics. However, long-term studies need to be done to compare it with different types of membranes.
